# The Hyr1 protein from the fungus *Candida albicans* is a cross kingdom immunotherapeutic target for *Acinetobacter* bacterial infection

**DOI:** 10.1371/journal.ppat.1007056

**Published:** 2018-05-10

**Authors:** Priya Uppuluri, Lin Lin, Abdullah Alqarihi, Guanpingsheng Luo, Eman G. Youssef, Sondus Alkhazraji, Nannette Y. Yount, Belal A. Ibrahim, Michael Anthony Bolaris, John E. Edwards, Marc Swidergall, Scott G. Filler, Michael R. Yeaman, Ashraf S. Ibrahim

**Affiliations:** 1 Department of Medicine, Los Angeles Biomedical Research Institute at Harbor-University of California at Los Angeles (UCLA) Medical Center, Torrance, California, United States of America; 2 Department of Medicine, David Geffen School of Medicine at UCLA, Los Angeles, California, United States of America; 3 Department of Biotechnology and Life Sciences, Faculty of Postgraduate Studies for Advanced Sciences (PSAS), Beni-Suef University, Beni-Suef, Egypt; 4 Portola High School, Irvine, California, United States of America; Geisel School of Medicine at Dartmouth, UNITED STATES

## Abstract

Different pathogens share similar medical settings and rely on similar virulence strategies to cause infections. We have previously applied 3-D computational modeling and bioinformatics to discover novel antigens that target more than one human pathogen. Active and passive immunization with the recombinant N-terminus of *Candida albicans* Hyr1 (rHyr1p-N) protect mice against lethal candidemia. Here we determine that Hyr1p shares homology with cell surface proteins of the multidrug resistant Gram negative bacterium, *Acinetobacter baumannii* including hemagglutinin (FhaB) and outer membrane protein A (OmpA). The *A*. *baumannii* OmpA binds to *C*. *albicans* Hyr1p, leading to a mixed species biofilm. Deletion of *HYR1*, or blocking of Hyr1p using polyclonal antibodies, significantly reduce *A*. *baumannii* binding to *C*. *albicans* hyphae. Furthermore, active vaccination with rHyr1p-N or passive immunization with polyclonal antibodies raised against specific peptide motifs of rHyr1p-N markedly improve survival of diabetic or neutropenic mice infected with *A*. *baumannii* bacteremia or pneumonia. Antibody raised against one particular peptide of the rHyr1p-N sequence (peptide 5) confers majority of the protection through blocking *A*. *baumannii* invasion of host cells and inducing death of the bacterium by a putative iron starvation mechanism. Anti-Hyr1 peptide 5 antibodies also mitigate *A*. *baumannii /C*. *albicans* mixed biofilm formation *in vitro*. Consistent with our bioinformatic analysis and structural modeling of Hyr1p, anti-Hyr1p peptide 5 antibodies bound to *A*. *baumannii* FhaB, OmpA, and an outer membrane siderophore binding protein. Our studies highlight the concept of cross-kingdom vaccine protection against high priority human pathogens such as *A*. *baumannii* and *C*. *albicans* that share similar ecological niches in immunocompromised patients.

## Author summary

Different pathogens share similar medical settings and rely on similar virulence strategies to cause infections. We have applied computational modeling and bioinformatics to discover novel antigens that target organisms sharing ecological niches in the intensive care units (ICUs): the fungus *Candida albicans*, and the multidrug resistant (MDR) Gram-negative bacterium, *Acinetobacter baumannii*. We identified that *C*. *albicans* hyphal wall protein Hyr1p shares significant structural homology to *A*. *baumannii* cell surface proteins, and is the receptor for *A*. *baumannii* binding to the fungus. Active vaccination (with rHyr1p) or passive immunization (anti-Hyr1p antibodies) protect mice from *A*. *baumannii* bacteremia and pneumonia. Anti-Hyr1p antibodies act synergistically with antibiotics in abrogating mixed species biofilms. Our groundbreaking studies reveal novel cross-kingdom immunotherapeutic strategies that target healthcare-associated MDR infections.

## Introduction

*Acinetobacter baumannii* has emerged as a frequent cause of healthcare-associated infections, ranging from bacteremia, pneumonia, urinary tract infections, to skin and wound infections, including those seen in the military theatre [1–10]. Its emergence in the healthcare environment is ascribed to its ubiquitous environmental presence, its ability to survive for prolonged periods of time on abiotic hospital surfaces, and its resistance to many existing antibiotics [11, 12]. Of great concern is the recent rise in the frequency of extensively drug resistant (XDR) *A*. *baumannii* infections, from fewer than 4% of all *A*. *baumannii* infections in 2000, to 60–70% in 2010 [2, 13–15]. Such XDR-*A*. *baumannii* infections often require treatment with second-line agents such as tigecycline and colistin, which are associated with clinical failure, development of pan-drug resistance and nephrotoxicity [16–26].

The recalcitrance of *Acinetobacter* to antibiotics is further exacerbated in the setting of biofilms [27, 28], which can promote its adherence to and colonization of indwelling devices such as urinary catheters and endotracheal tubes. [29] *A*. *baumannii* is particularly adept at forming polymicrobial biofilms in contexts of healthcare settings [30, 31]. Notably, *A*. *baumannii* often shares an ecological niche with the opportunistic pathogenic yeast, *Candida albicans*, especially in intensive care units [32–34]. In fact, these organisms represent two of the top five microorganisms associated with failure of endotracheal tube function, often due to biofilm formation [30]. Previous studies have also revealed complex *in vitro* and *in vivo* interactions between *C*. *albicans* and *A*. *baumannii*, in which *A*. *baumannii* may exploit *C*. *albicans* hyphae for adhesion [35, 36]. These interactions depend on an interplay between the *A*. *baumannii* protein OmpA, with an as yet unidentified receptor on *C*. *albicans*. *A*. *baumannii* also secretes molecules in growth media that inhibit fungal germination and hyphal formation. Interestingly, *C*. *albicans* combats the bacterium by producing quorum sensing molecule of its own—farnesol—and can dominate the shared niche when fungal density exceeds that of the bacterium [37]. This relationship suggests that a competitive dynamic exists between the two species in settings such as biofilms. The outcome of this interaction likely depends on multiple factors, including tissue or device affinity, immune evasion and secondary metabolite production. Interventions that interfere with colonization- or virulence-enabling interactions of the organisms, and/or which abrogate the formation or homeostasis of biofilms, could in concept be harnessed as therapeutic options.

We have previously applied computational modeling to discover novel antigen candidates that target more than one human pathogen [38]. This strategy is known as convergent or unnatural immunity, and has been demonstrated repeatedly in the development of viral and bacterial vaccines in which an antigen from a particular organism protects against another pathogen from the same kingdom [38]. Cross-kingdom protective antigens have also been reported, in which an antigen from a source organism protects against organism(s) from other biological kingdoms [39–41]. Specifically, we identified unforeseen 3-D structural and functional homology between members of the Agglutinin like sequence (Als) family of proteins of *C*. *albicans* that induce adhesion to and invasion of mammalian cells, and surface adhesin/invasin molecules (MSCRAMMs) of *Staphylococcus aureus* [42]. Indeed, a recombinant form of the Als3 protein (currently in clinical trials) elicits robust T- and B-cell responses and protects mice from both *Candida* and *S*. *aureus* infections [39–41, 43–46]. Of considerable interest is our current discovery that the *C*. *albicans* hyphal cell surface glycosylphosphatidylinositol (GPI)-anchored protein Hyr1p is predicted to have 3-D structural and epitope homology to known and putative virulence factors of specific Gram-negative bacteria including *A*. *baumannii*. Thus, we pursued studies to examine the potential use of rHyr1p as a vaccine candidate to mediate protection against *A*. *baumannii* in mice using the FDA-approved alum as an adjuvant. We further sought to evaluate the potential use of passive immunotherapy against *A*. *baumannii* using anti-Hyr1p antibodies, and investigate the functionality of these specific antibodies in abrogating *Acinetobacter* virulence *in vitro* and in a mixed species biofilm with *C*. *albicans*.

## Results

### Hyr1p is structurally related to microbial adhesion proteins of *A*. *baumannii*

Bioinformatic, homology and energy-based modeling identified a number of conserved physicochemical structural domains within Hyr1p and several Gram-negative antigens. In particular, these algorithms identified multiple β-helical structures composed of parallel β-strands that typically create either two or three faces along within the holoprotein structure. Examples of β-helical templates identified included the phage φAB6 tailspike protein, which specifically binds oligosaccharides on the *A*. *baumannii* surface [47], the *Haemophilus influenzae* high molecular weight (HMW1) adhesin, and the filamentous hemagglutinin adhesin (FhaB) from *Bordetella pertussis*. Notably, FhaB has also been identified as an outer membrane protein of *A*. *baumannii* and is considered a protective immunogen [48].

**Fig 1:**
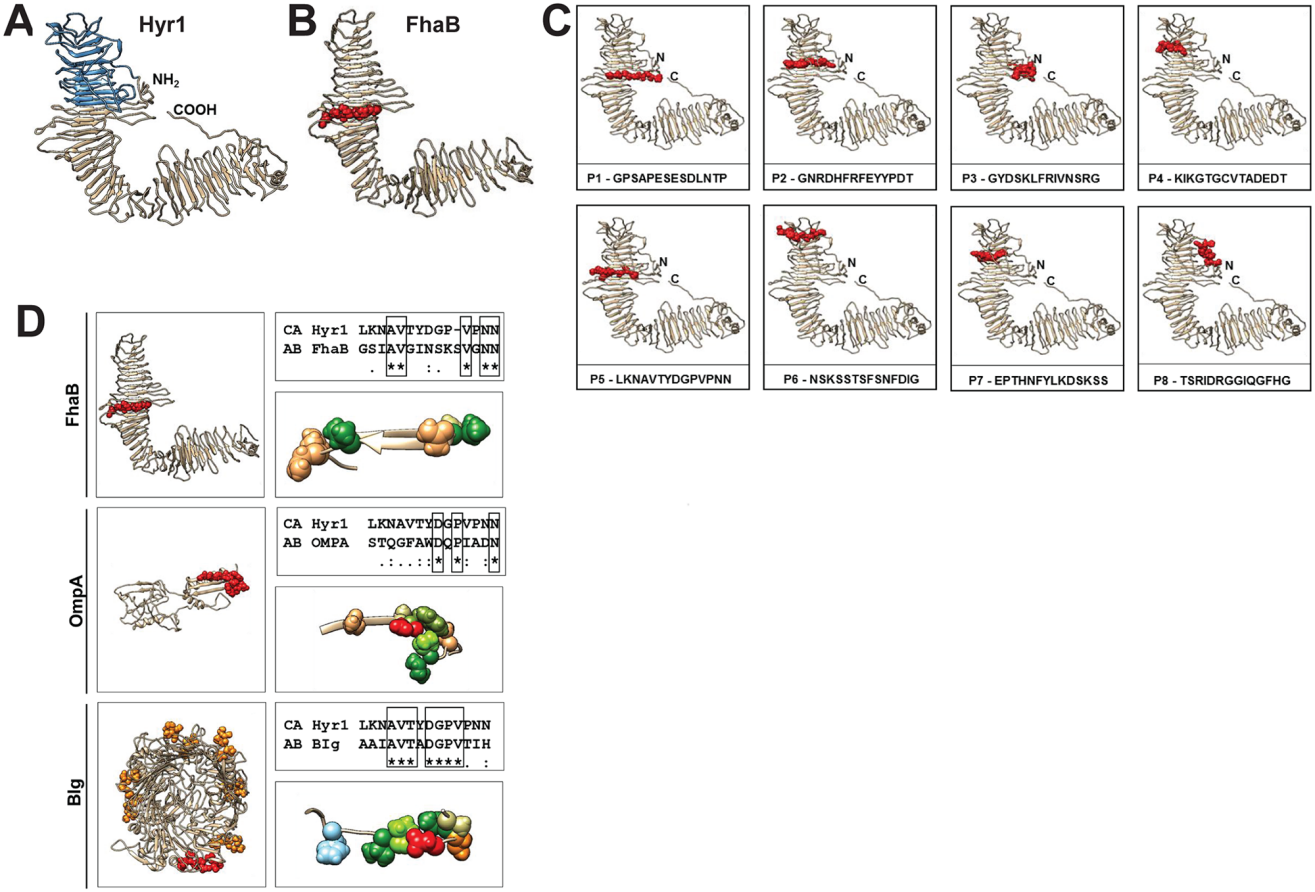
Striking 3 D structural homology between *C*. *albicans* Hyr1p and *A*. *baumannii* FhaB. Ribbon diagram depicting the full scale model for *C*. *albicans* Hyr1 as computed by the iTasser server (A). The region of Hyr1 corresponding to homologous domains in the viral tailspike, *BP* FhaB and *HI* HMW1 proteins is indicated in blue. Note the striking similarity in 3D structure of Hyr1p and FhaBp (B). Hyr1 models showing the sequence and location of the eight peptides used as antigens in the study in red as van der Waals space-filling spheres (C). Note the similar location of peptide 5 on Fhab (B) and on Hyr1p (C) (red regions). Models for three putative cross-reactive *A*. *baumannii* targets (FhaBp, OmpA, BIg) are shown with domains homologous to peptide 5 in red (D). In addition, the *A*. *baumannii* BIg protein has many additional near matches (6 identical residues) which are shown on the full model as orange. Sequence alignments between *C*. *albicans* (CA) peptide 5 and putative cross-reactive *A*. *baumannii* (AB) targets with identical residues are depicted in boxes. Higher resolution views of identified domains showing identical and physicochemically conserved residues are visualized as space-filling spheres. Coloration is a modified RasMol schema (Gly, Ala—cream; Asn, Gln—light brown; Thr—orange; Val, Ile—green; Trp—olive green; Asp—red; His—sky blue; Pro—chartreuse).

Once the above domains were identified, they, along with other high scoring templates, were assembled and subjected to energy minimization and hydrogen-bond optimization to ultimately produce 3-D structural models for analysis in relation to Hyr1p (iTasser model shown; Fig 1A). The resulting structure is largely β-helical with limited unstructured and extended domains. Based on this modeling strategy, *C*. *albicans* Hyr1p shares striking similarity to *A*. *baumannii* FhaB protein (Fig 1B), and contains motifs that appear to be common to other target proteins expressed by this bacterium.

### Antibodies raised against Hyr1 peptides specifically recognize *A*. *baumannii*

**Fig 2:**
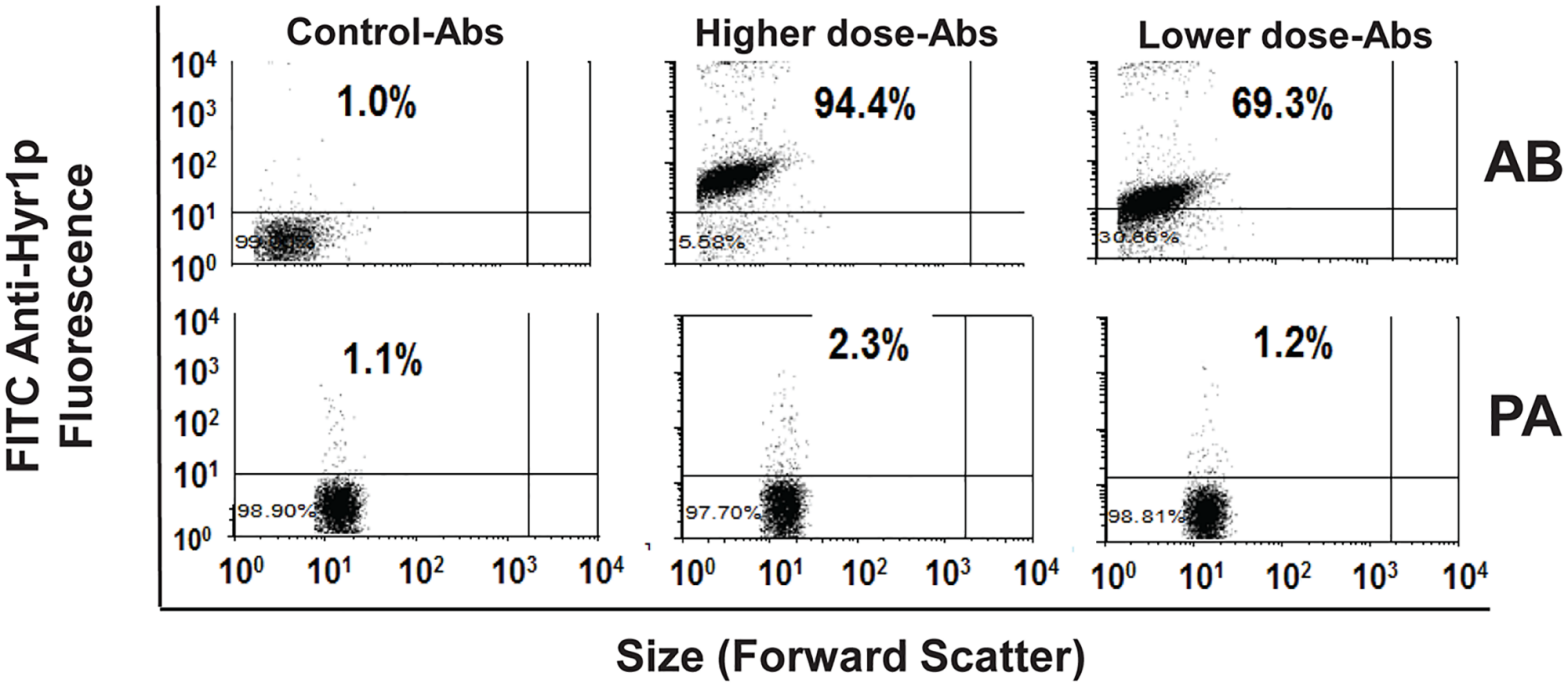
Anti-Hyr1p peptide antibodies specifically binds *A*. *baumannii* but not *P*. *aeruginosa*. Flow cytometry analysis of pooled sera collected from rabbits separately immunized with 8 peptides of Hyr1p showing binding of these antibodies to *A*. *baumannii* but not to *P*. *aeruginosa* (bacterial cells at 10^7^ cells). Pooled sera from the same rabbits prior to immunization was used as a control (Control-Abs). The high and the low doses of the Abs were 30 and 3 μg/ml, respectively. *AB = A*. *baumannii*, *PA = P*. *aeruginosa*.

To validate the results of our bioinformatics analysis, we raised rabbit polyclonal antibodies against 8 peptides of Hyr1p-N that were predicted to be surface exposed and highly antigenic [49]. These antibodies specifically recognized *C*. *albicans* hyphae *in vitro* and protected mice from hematogenously disseminated candidiasis [50]. The location of the eight peptide sequences on the 3-D structural model for Hyr1p are indicated in Fig 1C. To investigate the specificity of the anti-Hyr1p antibodies to *Acinetobacter*, we compared their binding capacity to *A*. *baumannii* vs. *Pseudomonas aeruginosa* (which contains surface proteins predicted to have low homology to Hyr1p). Using flow cytometry and immunostaining, the antibodies raised against the Hyr1 8 peptides targeted log phase cells of *A*. *baumannii* (69–95%) (Fig 2). In contrast, the same antibodies did not bind to *P*. *aeruginosa* (Fig 2). We also tested the ability of these antibodies to bind to several clinical isolates of XDR-*A*. *baumannii* with clonal variability [51]. The antibodies bound to all tested isolates, indicating that this recognition is not strain specific (S1 Fig). These results demonstrate the specificity of anti-Hyr1p antibodies to *A*. *baumannii* and further validate the modeling strategy which revealed these unforeseen homologies and epitopes.

### *A*. *baumannii* binds to *C*. *albicans* via Hyr1

**Fig 3:**
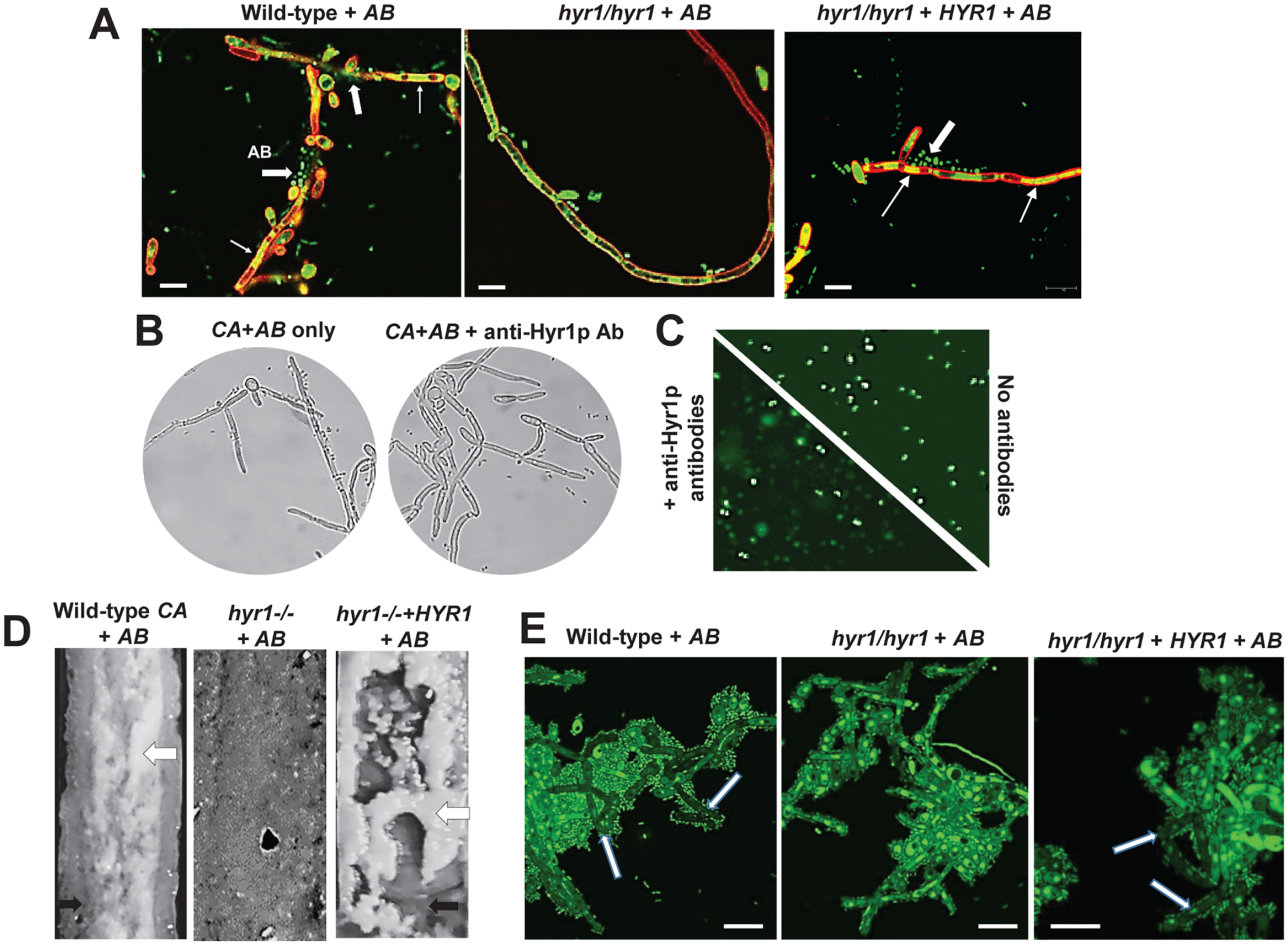
Hyr1 is the *C*. *albicans* receptor for *A*. *baumannii* binding to hyphae, and attachment of *A*. *baumannii* to *C*. *albicans* in a flow biofilm model requires Hyr1p. *A*. *baumannii* (*AB*) and *C*. *albicans* (*CA*) cells were allowed to interact physically under hyphae permissive conditions. The cells were stained with ConA (*Candida* cell wall red) and Syto13 (nuclei green). *Acinetobacter* binds to (block arrows), and kill wild-type or *hyr1/hyr1+HYR1* complemented *C*. *albicans* hyphae as observed by degraded fungal nuclei (thin arrows) (A). The bacteria cease to attach to and kill hyphae of *C*. *albicans hyr1/hyr1* mutant strain (A). Anti-Hyr1p-N antibodies abolish attachment of the bacteria to the fungal filaments (B) and inhibit *Acinetobacter* viability (diminished nuclear staining = dead cells in presence of antibody) (C). Under flow mixed-species biofilm conditions, *Acinetobacter* binds to wild-type or *hyr1/hyr1+HYR1* complemented *C*. *albicans* biofilm (black arrow) and forms a thick white layer (white arrow), whereas the bacterial binding is considerably reduced on the *C*. *albicans hyr1/hyr1* mutant cells (D). The extent of *A*. *baumannii* binding to *C*. *albicans* wild-type, *hyr1/hyr1*, or *hyr1/hyr1+HYR1* complemented strains is microscopically depicted, where the cells are stained with Syto13. Note the dead hyphae in the wild-type and *hyr1/hyr1+HYR1* complemented strains but not in the *hyr1/hyr1* mutant (white arrows) (E). Bars in A and E are 10 μm.

We co-cultured *Acinetobacter* and *Candida* under planktonic, hyphal growth-permissive conditions. We found that *Acinetobacter* bound robustly to wild-type or *hyr1/hyr1 + HYR1* complemented *C*. *albicans* hyphae, prevented elongation, and killed it within 4 h of co-culture as determined by Syto 13 nuclear staining showing fragmented nuclei (Fig 3A). In contrast, a *C*. *albicans hyr1/hyr1* mutant hyphae had minimal *A*. *baumannii* attachment and an elongated phenotype harboring intact nuclei (Fig 3A). The role of Hyr1p as a receptor for *Acinetobacter* was verified by blocking Hyr1p function with anti-Hyr1p-N antibodies. These antibodies blocked bacterial attachment to the fungal hyphal cells (Fig 3B), indicating that Hyr1p is a *C*. *albicans* receptor for *A*. *baumannii*. Furthermore, we found that at 24 h incubation with the 100 μg/ml antibodies the viability of *A*. *baumannii* decreased by 39% ± 4% when compared to bacteria incubated in the same media without the antibodies (Fig 3C, *P* <0.05).

We also found that cell-free filtrates of an overnight culture of *A*. *baumannii* prevents *C*. *albicans* filamentation and biofilm formation in a static 96 well microtiter plates (S2A Fig), indicating that *A*. *baumannii* inhibits *C*. *albicans* biofilm formation by secreted substance(s) that are yet to be identified. To minimize the effect of quorum sensing molecules secreted by both organisms, we studied the interactions of *C*. *albicans* with *A*. *baumannii* in a mixed species biofilm model in which there is a continuous flow of fresh medium. Indeed, in the flow system, *C*. *albicans* had higher viability when mixed with *A*. *baumannii* than in a static model (S2B Fig).

Under conditions of flow, and after 24 h of biofilm development, *Acinetobacter* bound to wild-type or *hyr1/hyr1 + HYR1* complemented *C*. *albicans* hyphae and developed a visibly thick, white layer of bacterial cells (Fig 3D). Mixed species biofilms formed with the *C*. *albicans hyr1/hyr1* mutant did not display such a robust attachment of the bacteria to the fungi. Aliquots of the two respective biofilms, when stained with Syto13 and observed under the microscope, revealed enhanced attachment of bacterial cells to the wild-type or *hyr1/hyr1 + HYR1* complemented fungal filaments, where the bacteria clustered together to fill up spaces between the hyphal cells (Fig 3E). Nuclear staining additionally revealed that the wild-type or *hyr1/hyr1 + HYR1 complemented* cells were largely non-viable, displaying dark hyphae with degraded nuclei (depicted by the white arrow in Fig 3E). On the other hand, there was a significant reduction in the attachment of *A*. *baumannii* to the *C*. *albicans hyr1/hyr1* hyphae, with the cells exhibiting an overall intact nuclear stain and no dark hyphae (Fig 3E). While there was a stark reduction in the numbers of bacteria attached to the *hyr1/hyr1* mutant, the hyphae were not completely free of bacterial cells, suggesting receptors other than Hyr1p also contribute to this interaction. Als3p is a key *C*. *albicans* hyphal wall adhesin with roles in promoting attachment of other Gram negative bacteria such as *P*. *aeruginosa*, to hyphae [52]. Thus, we tested *C*. *albicans als3/als3* mutant in interacting with *A*. *baumannii*. *A*. *baumannii* adhered robustly to *als3/als3* filaments, just like the wild-type filaments (S3 Fig).

### Identification of *A*. *baumannii* proteins that bind to *C*. *albicans* Hyr1p

**Fig 4:**
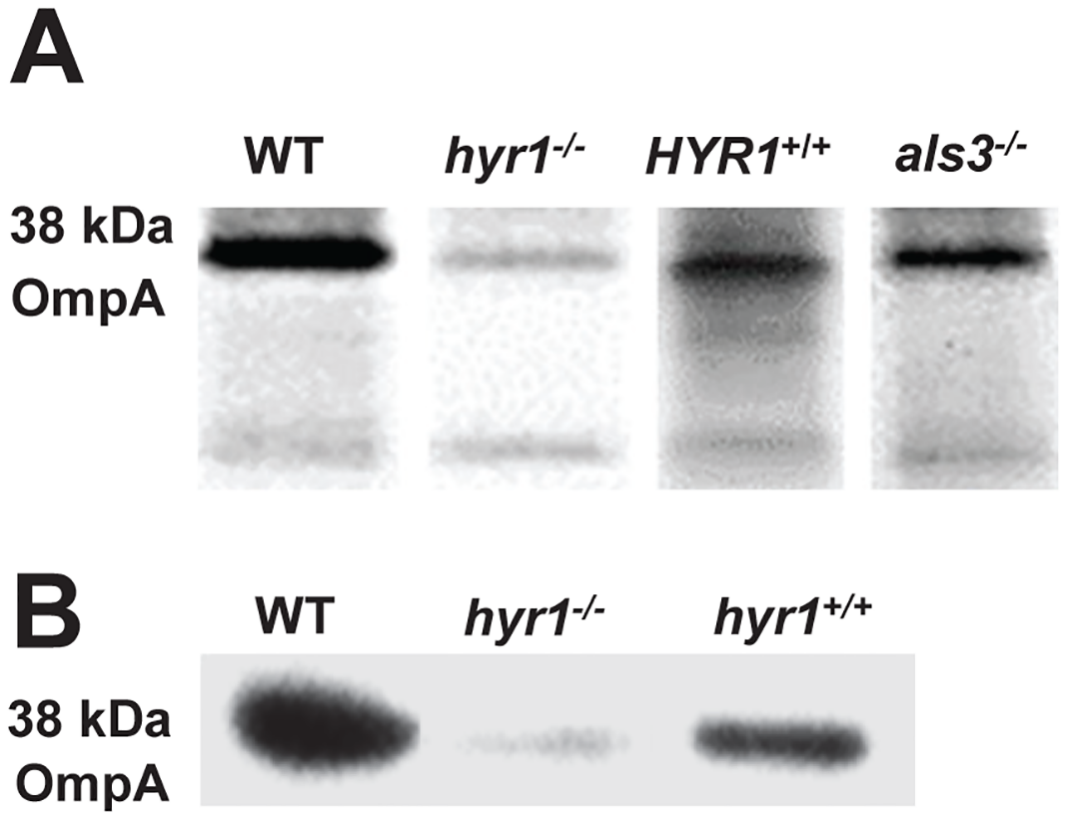
*Acinetobacter* OmpA is the ligand to *Candida* Hyr1p. *Acinetobacter* biotin-labeled cell wall proteins that bound to *C*. *albicans* wild-type, *hyr1/hyr1*, *hyr1/hyr1+HYR1*, or *als3/als3* hyphae were separated on SDS-PAGE, probed with anti-biotin antibody, and the hybridized bands were identified by Mass spectrometry. *C*. *albicans* wild-type or *hyr1/hyr1+HYR1* complemented hyphae bound a major band at 38 kDa which was identified as OmpA by MS/MS analysis (A). In contrast, the *hyr1/hyr1* mutant had severe reduction in its ability to bind OmpA. *C*. *albicans* Als3p was not found to be a receptor as proteins equally bound to *als3/als3* mutant strain (A). To confirm the identity of the 38 kDa band, blots were probed with an anti-OmpA antibody, which confirmed that the 38 kDa proteins were indeed OmpA (B).

To identify the complimentary Hyr1p ligand on the bacterium that mediates binding of *A*. *baumannii* to *C*. *albicans*, we performed affinity purification of biotin-labeled *A*. *baumannii* cell membrane proteins using *C*. *albicans* hyphae. The following *C*. *albicans* strains were used for this interaction: wild-type, *hyr1/hyr1* homozygous mutant, *hyr1/hyr1* + *HYR1* complemented strain, and *als3/als3* as an additional control. *A*. *baumannii* protein bands that bound to different *C*. *albicans* hyphae were visualized on Western blot using anti-biotin antibodies. When incubated with membrane extracts of *A*. *baumannii*, *C*. *albicans* wild-type, *hyr1/hyr1* + *HYR1* complemented strain, or *als3/als3* mutant hyphae bound a major band at 38 kDa. This band was largely absent when the cell membrane proteins were affinity purified with the *hyr1/hry1* mutant (Fig 4A). The mass of the 38 kDa band was equivalent to OmpA, and OmpA was reported to be an *A*. *baumannii* receptor for *C*. *albicans* hyphae [35]. Therefore, we repeated the affinity purification studies using anti-OmpA antibodies. As expected, this 38 kDa band was strongly bound by OmpA antibodies in the wild-type strain, but little or no binding was observed in the *hyr1/hyr1* mutant (Fig 4B). Finally, the identity of the band as OmpA was confirmed by using LC-MS. Thus, *A*. *baumannii* binds to *C*. *albicans* hyphae via OmpA/Hyr1p interactions.

### Active vaccination using rHyr1p-N protectes mice from *A*. *baumannii* infection

**Fig 5:**
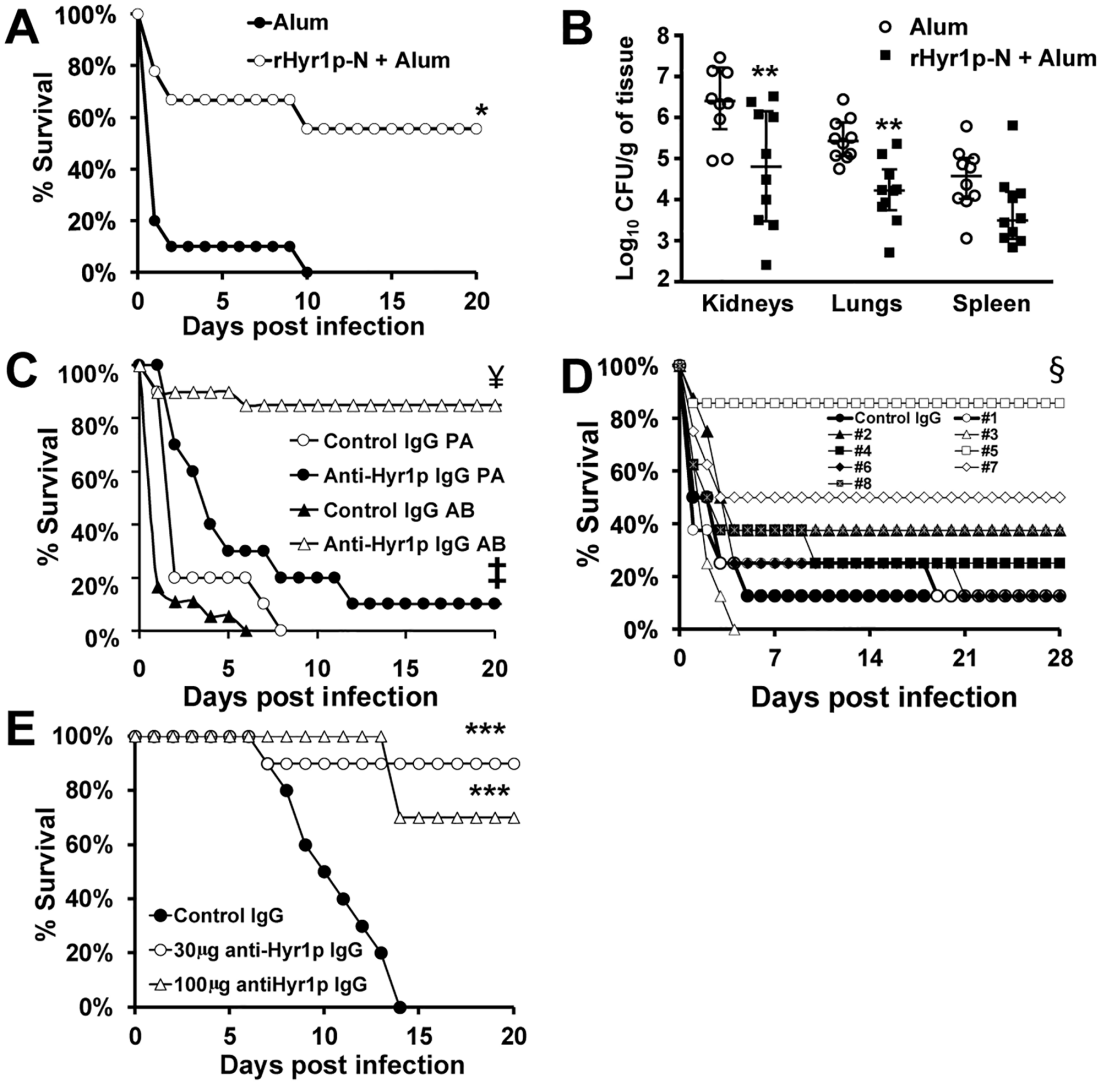
Active or passive immunization targeting Hyr1p protect diabetic and neutropenic mice from *A*. *baumannii* infections. Survival of diabetic mice (n = 10 for alum control or 9 for rHyr1p-N + Alum) were vaccinated and boosted with 30 μg dose mixed with 0.1% aluminum hydroxide then infected with *A*. *baumannii* HUMC1 (confirmed inoculum of 2.5 x 10^7^) via the tail vein. *P = 0.005 vs. alum control (A). Tissue bacterial burden of target organs harvested from diabetic mice (n = 10 per arm) vaccinated with alum or rHyr1p + alum three days after infecting with *A*. *baumannii* (5 x 10^6^). **P <0.02 vs. alum vaccinated mice (B). Survival of diabetic mice that were given an intraperitoneal (i.p.) injection of 1 mg of pooled anti-Hyr1p IgG raised against 8 individual peptides of Hyr1p (n = 20 from 2 independent experiments with similar results for *A*. *baumannii* [AB] infection and 10 for *P*. *aeruginosa* [P*A*]) or control IgG (n = 18 for AB and 10 for PA) 2 hours prior to infecting them intravenously (i.v.) with AB or PA (confirmed inocula of 3.4 x10^7^ cells for AB and 1.1 x 10^8^ for PA). ^¥^*P* = 0.00001 vs. Control IgG of AB ^‡^*P* = 0.1 vs. Control IgG of PA (C). Survival of diabetic mice (n = 8 per group) that were given an i.p. injection of 1 mg of each anti-Hyr1p IgG raised against individual peptides of Hyr1p 2 hours prior to infecting them i.v. with *A*. *baumannii* (confirmed inoculum of 6.2 x 10^7^). ^§^*P <*0.05 vs. all arms except peptide, 2, 7, or 8 (D). Survival of neutropenic mice (n = 10 per group) infected with *A*. *baumannii* via inhalation (inhaled inoculum of 2.2 x 10^7^) and 24 h later treated i.p. with either isotype matching control IgG (100 μg), or with anti-peptide 5 IgG (at 30 or 100 μg). A repeat dose of the antibody was given on Day +8. ***P <0.002 vs. Control IgG arm (E).

To explore its potential as a cross-kingdom protective antigen, we tested Hyr1p as an active vaccine target against *A*. *baumannii* infections *in vivo*. Mice were subcutaneously vaccinated on day 0, with rHyr1p-N mixed with alum [49, 53], boosted with a similar dose on day 21, and then infected with a lethal dose of XDR *A*. *baumannii* HUMC1 via i.v. injection on day 35 after making them diabetic. Diabetic mice were used because normal mice are resistant to infection, and diabetes is a risk factor for developing *A*. *baumannii* infections [51, 54]. Consistent with our i.v. model [51], the alum control mice had almost 100% mortality by day 2 post infection, while ~60% of the mice receiving the vaccine survived the infection even after 20 days (Fig 5A). Surviving mice had no bacteria detected in their organs at day 21 and appeared healthy. Corroborating this protective effect, kidneys and lungs harvested as early as 3 days post infection from rHyr1p-N-vaccinated mice had >1 log reduction in their bacterial burden when compared to tissues harvested from mice vaccinated with alum alone (Fig 5B). Further, the bacterial burden of spleen taken from rHyr1p-N-vaccinated mice strongly trended to be a log less than the spleen from alum vaccinated mice (*P* = 0.05) (Fig 5B).

### Passive immunization using anti-Hyr1 antibodies protectes against *A*. *baumannii* infection

Next, we tested the efficacy of pooled purified IgG raised against the eight 14-mer peptides predicted to be surface exposed on Hyr1p-N (Fig 1C) [53]. Diabetic mice that prophylactically received the anti-Hyr1p IgG were almost completely protected from *Acinetobacter* bacteremia when compared to mice receiving isotype matching control antibody (85% survival for anti-Hyr1p antibodies treated mice vs. 0% for isotype matching control IgG) (Fig 5C). Of note, this protection was specific since anti-Hyr1p IgG did not protect mice from *P*. *aeruginosa* infection (Fig 5C, p = 0.1), which has no proteins that are predicted to share homology with Hyr1p and bound poorly to Hyr1p antibodies (Fig 2).

We further investigated which peptide-targeting antibodies were responsible for conferring the majority of protection. Diabetic mice were prophylactically treated individually with 8 different antibodies raised against the peptides (see Fig 1C for the location of the peptide on the modelled Hyr1p). Purified IgG from each of the generated polyclonal antibodies was administered 2 h prior to infecting the mice with a lethal dose of *A*. *baumannii* via tail vein injection. We found that Anti-Hyr1p IgG raised against peptide #5 (LKNAVTYDGPVPNN) [53] protected mice from infection similarly to the combined antiHyr1p IgG pool (i.e. 85% survival) (Fig 5D). These results indicate that the protection conferred by the anti-Hyr1p IgG is largely conferred by peptide 5 in cross protection against *Acinetobacter* infection.

Becuase penumonia is a major manifestation of the disease and commonly seen in severly immunosuppressed patients, we tested the ability of anti-peptide 5 antiboides to therapeutically treat *A*. *baumannii* penumonia in neutropenic mice. Immunosuppresed mice were infected with *A*. *baumanni* cells via inhalation and 24 h later were treated with doses of 100 or 30 μg of anti-peptide 5 purified IgG (established infection). Mice that received a 100 μg of isotype matching control IgG had 100% mortaility by day 14. In contrast, mice reciving 100 μg or 30 μg doses of anti-peptide 5 antibodies had 70% or 90% long-term survival, respectively (Fig 5E). Surviving mice appeared healthy by day 20 when the expeirment was terminated. Additionally, surviving mice had no residual bacterial burden in their lungs as determined by quantitative culturing. These low doses of curative antibodies confirm their specific protection meachnism and their translational potential as a novel treatment for *A*. *baumannii* bacteremia and penumonia in different hosts (diabetics and neutropenics).

### Anti-peptide 5 antibodies recognize cell surface antigens of *A*. *baumannii*

**Fig 6:**
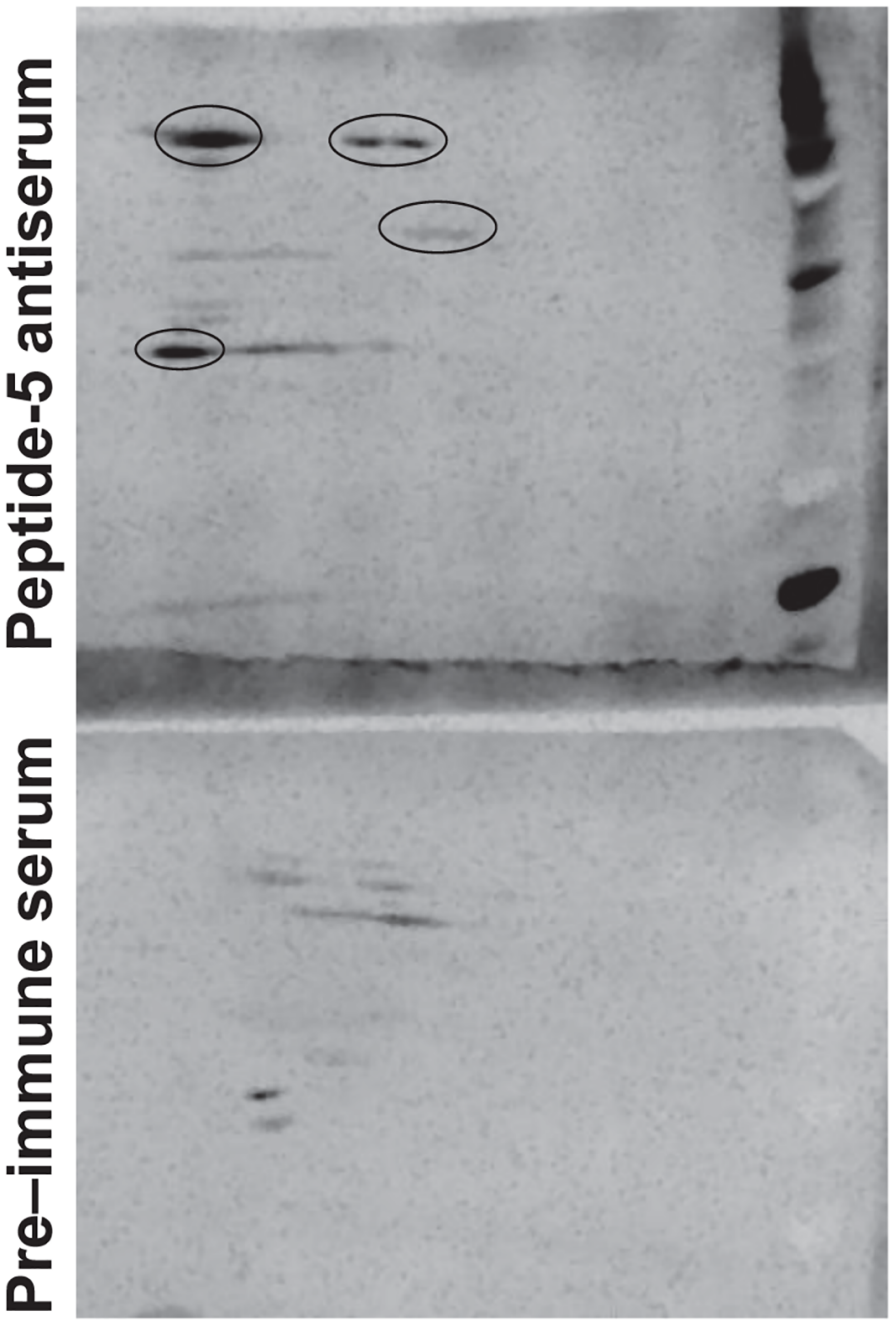
Anti-peptide 5 serum recognizes four unique *A*. *baumannii* cell surface proteins. Cell surface proteins of XDR *A*. *baumannii* HUMC1 were separated on a 2D gel and western blot was carried out using serum from rabbits immunized with Hyr1p peptide #5 antigen. Serum from the same rabbit prior to immunization was used as a control (pre-immune serum). Notice the four spots (circles) recognized by the immune serum vs. the pre-immune serum.

Because anti-peptide 5 antibodies protected mice from *Acinetobacter* infections, we tested their ability to recognize *A*. *baumannii* cross-reactive cell membrane antigens using high resolution 2-D Western blotting followed by MALDI-TOF-MS/MS analysis. Rabbit anti-peptide-5 antisera intensely recognized four protein spots as compared to pre-immune serum collected from the same rabbit (Fig 6). These spots were identified as OmpA, a putative ferric siderophore outer membrane binding protein (TonB-dependent), a putative outer membrane protein, and FhaBp.

As a complementary approach, we initiated a bioinformatics search to identify potential cross-reactive antigens homologous to peptide 5 in *A*. *baumannii*. Several such domains were identified and characterized in FhaBp and OmpA proteins of *A*. *baumannii*. Moreover, we identified the *Acinetobacter* Bacterial Immunoglobulin-like domain (BIg) family of proteins as having significant identity with the peptide 5 sequence (7/8 [88%] motif identity; 7/14 [50%] identity over the 14 residue span). Models of these domains were generated with identical and functionally conserved residues visualized as van der Waals space-filling spheres (Fig 1D).

### Anti-peptide 5 serum blocks *A*. *baumannii*-mediated invasion of host cells *in vitro* and is bactericidal likely by an iron starvation mechanism

**Fig 7:**
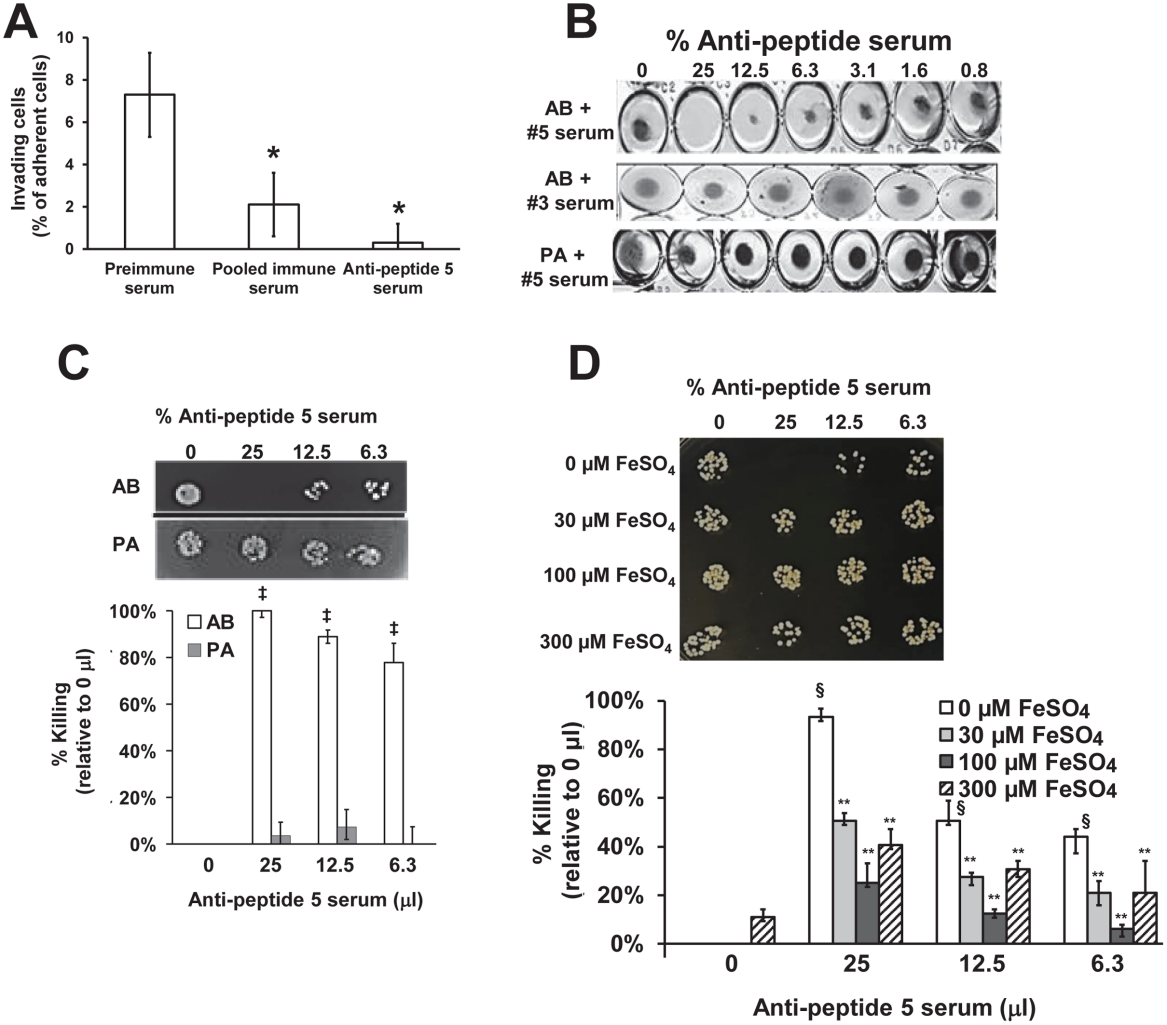
Invasion of alveolar epithelial cells by XDR *A*. *baumannii* in the presence of rabbit serum collected before or after immunization (10 μg) with Hyr1p peptides. Data (n = 6) expressed as % invading cells (mean + SD) of the adherent cells enumerated after lysing mammalian cells without colistin treatment (A). *P<0.01 vs. preimmune serum. Effect of anti-peptide 5 serum on the viability of *A*. *baumannii* (*AB*) or *P*. *aeruginosa* (*PA) in vitro* (B). Bacterial cells (1 x 10^5^ cells) in MHII medium were incubated in 96-well plate at 37°C for 20 h with varying concentrations of the immune serum. Killing activity of anti-peptide 5 serum was enumerated by CFU (n = 3 per arm) expressed as % killing relative to cells without any anti-peptide 5 serum. ^‡^*P*<0.005 vs. no serum (C). Experiments in B were repeated with *A*. *baumannii* inoculated in different concentrations of FeSO_4_ supplemented media (D) and the CFU (n = 6 per arm) were enumerated and expressed as % killing relative to cells without any anti-peptide 5 serum or FeSO_4_. ^§^P< 0.01 vs. no serum no FeSO_4_. **P <0.05 vs. no FeSO_4_ at the corresponding serum concentration.

Next, we set out to explain the protective mechanisms elicited by Hyr1p as an immunogen against *A*. *baumannii*. Due to the cross reactivity and homology of Hyr1p to FhaBp, and OmpA (known adhesins/invasins in *A*. *baumannii* [55–57]) we reasoned that the anti-Hyr1p antibodies may interfere with the ability of *Acinetobacter* to adhere to and/or invade host cells. We chose to investigate the effect of anti-peptide 5 antibodies on the ability of *A*. *baumannii* to invade human alveolar epithelial cell line (A549) since pneumonia is a common manifestation of this bacterial infection. The pooled sera raised against the 8 peptides reduced the ability of *A*. *baumannii* to invade alveolar epithelial cells by ~70%, while sera raised against peptide 5 almost completely blocked invasion when compared to pre-immune sera (Fig 7A).

As mentioned earlier, the antibodies raised against 8 different peptides of Hyr1p, displayed inhibitory effect on *A*. *baumannii* viability when incubated with the bacterium for longer periods (e.g. ≥20 h, Fig 3C). To expand on this observation, we tested if the protective anti-peptide 5 serum would display similar or enhanced killing of the bacteria. Indeed, we found that anti-peptide 5 serum, but not anti-peptide 3 serum [which did not protect mice from *A*. *baumannii* infection (Fig 5D)], inhibited the growth of *Acinetobacter in vitro* when incubated with the bacterium for 20 h. The antiserum did not inhibit the growth of *P*. *aeruginosa* (Fig 7B). Further, we determined that this growth inhibition of *A*. *baumannii* by anti-peptide 5 serum was bactericidal (Fig 7C). The killing activity of the anti-peptide 5 serum was not abrogated by heating the serum at 60°C for 60 min prior to incubating with the bacteria. Collectively, these results support the specificity of the anti-peptide 5 antibodies in protecting against *A*. *baumannii* infection, and demonstrate that the growth inhibition of the anti-peptide 5 antibodies is independent of complement.

Our Western blotting analysis using anti-peptide 5 serum (Fig 6) showed that a siderophore outer membrane binding protein (TonB-dependent) is a candidate cross-reactive antigen to Hyr1p. Also, our structural homology modeling as well as Western blotting studies implicated OmpA as another antigen that is likely to cross react to Hyr1p antibodies. Both proteins are involved in iron acquisition [58, 59]. Therefore, we hypothesized that the antibodies might exert their inhibitory effect by iron starvation. Thus, we tested the inhibitory effect of anti-peptide 5 serum in the presence or absence of different concentrations of iron supplementation. Addition of iron in the form of FeSO_4_ reversed the bactericidal activity of the antibodies. This reversal was concentration dependent with 30 μM FeSO_4_ showing around 50%-80% reversal of the serum inhibitory effect while 100 μM FeSO_4_ had almost complete reversal of the bactericidal activity. Consistent with the toxic effect of higher iron concentrations [60], FeSO_4_ at 300 μM had less degree of reversal of the bactericidal activity of anti-peptide 5 serum (Fig 7D). These results indicate that the cidal activity of the anti-peptide 5 serum is due, at least in part, to blocking the ability of the bacterium in acquiring iron.

### Anti-peptide 5 serum is synergistic with antibiotics against *A*. *baumannii*

**Fig 8:**
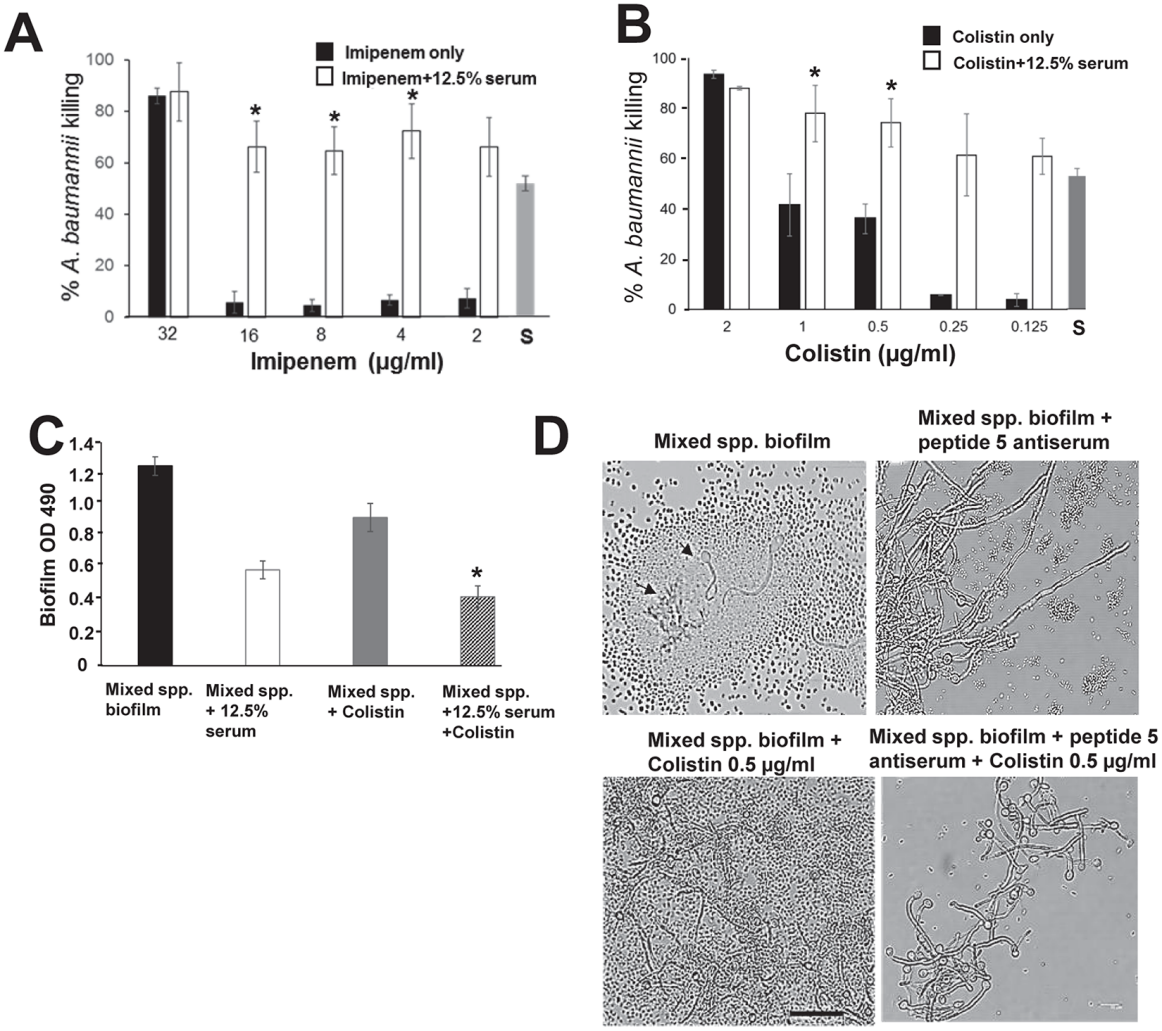
Anti-peptide 5 serum is synergistic with antimicrobials, against biofilms. *A*. *baumannii* were grown in the presence of imipenem (2–32 μg/ml, x axis) without anti-peptide 5 serum (black bars) or in combination with 12.5% of the anti-peptide 5 serum (white bars) (A). The same assay was performed with different concentrations of colistin (0.125–2 μg/ml) (B). S in the A and B denotes 12.5% anti-peptide 5 serum only. *P <0.02 vs. S. Mixed species biofilms were treated with 0.5 μg colistin, 12.5% serum or a combination of colistin and serum. Metabolic activity of the biofilm cells was measured by the XTT assay (OD 490 nm) (C). *P <0.05 vs. all other three conditions. Mixed species biofilm architecture under the four conditions in panel C were visualized by light microscopy (D).

Given that anti-peptide 5 antibodies have significant inhibitory activity against *A*. *baumannii* growth, we questioned if it could make this XDR organism more susceptible to antibiotics. We chose two drugs for the combination studies: 1) imipenem is a carbapenem often used as first line therapy to treat *A*. *baumannii* infection; and 2) colistin is an antibiotic often used as a last resort for treatment of XDR *A*. *baumannii* infections. The HUMC1 study strain (an XDR clinical isolate) exhibited a 50% inhibitory concentration (IC_50_) of imipenem at 32 μg/ml per CLSI method. Colistin exhibited an IC_50_ of 2 μg/ml against this *A*. *baumannii* strain. Likewise, the IC_50_ of the anti-serum against the test inoculum was a 12.5% dilution (vol/vol). When the antibodies were combined with serial dilutions of imipenem, we observed a significant synergistic effect in which the IC_50_ was reduced from 32 to 4 μl/ml (Fig 8A). A modest but additive effect was observed with antisera combined with colistin, lowering the IC_50_ to 0.5 or 1.0 μg/ml when compared to either colistin or the anti-peptide serum alone (Fig 8B). Control pre-vaccinated serum at the identical dilution did not produce this inhibition effect. We further confirmed these results by conducting time kill assays using sub-inhibitory concentrations of colistin at 0.5, 0.25, and 0.125 MIC with or without serum (at 12.5%). Clear and significant synergistic effect was noticed in killing *A*. *baumannii* when the anti-peptide serum was combined with the sub-inhibitory colistin concentrations especially after 24 h of incubation resulting in 50–90% reduction of bacterial count when compared to colistin or anti-peptide serum alone with the highest effect seen with serum combined with the 0.5 MIC colistin (S3 Fig).

### Anti-peptide-5 antiserum is synergistic with antibiotics vs. *A*. *baumannii* in mixed biofilms with *C*. *albicans*

*A*. *baumannii* commonly occupies shared niches with *C*. *albicans* in patients, and the two organisms can form mixed species biofilms [61, 62]. Thus, we studied the inhibitory effect of the anti-Hyr1 antibodies in controlling *A*. *baumannii/C*. *albicans* in a mixed biofilm model with or without colistin. The two organisms were grown together for 6 h to initiate development of biofilm. Anti-peptide 5 serum diluted to 12.5% (as above) was added to the mixed species biofilm with or without 0.5 μg/ml colistin, and the biofilms were allowed to evolve for another 12 h. By XTT quantification, in the presence of 12.5% anti-peptide 5 antiserum or colistin alone, mixed species biofilms displayed inhibitions of 50% or 30%, respectively. When the biofilms were treated with a combination of colistin and serum, an inhibition of ~70% was observed (Fig 8C). The inhibitory effect of anti-peptide serum combined with colistin is evident in micrographs taken from each individual culture showing decimation of bacterial growth without affecting *Candida* viability and growth (Fig 8D). These results suggest that immunotherapeutic strategies targeting Hyr1p can act additively with antibiotics to significantly reduce a complex *A*. *baumannii/C*. *albicans* biofilm formation.

## Discussion

Healthcare-associated infections are often caused by commensal organisms. These organisms frequently occupy shared host niches and exploit common vulnerabilities in the compromised host. In the setting of immune deficiency, such commensals exert similar virulence mechanisms (e.g. adherence to and invasion of host cells, iron acquisition, immune avoidance) to cause disease. It is logical that such organisms employ common virulence factors structurally and functionally evolved to be convergent in their interaction with the host. In turn, it is highly likely that the host has devised countermeasure defense strategies to recognize and protect against such infections. We hypothesize that such adapted host defense mechanisms can be harnessed for the development of novel immunotherapeutic strategies [38].

We have applied innovative computational molecular modeling and bioinformatic strategies to discover novel vaccine antigen candidates that target more than one high priority human pathogens. This strategy to identify convergent antigens has been validated in cross-kingdom immuno-protection against *C*. *albicans* and *S*. *aureus* [39, 40], in which the *C*. *albicans* adhesins/invasins Als family of proteins share structural and functional homology with MSCRAMMs of *S*. *aureus* (e.g. clumping factor A) [42]. Herein, we used this strategy to identify significant 3-D structural homologies among *C*. *albicans* Hyr1p and several candidate antigens of the XDR *A*. *baumannii*, two organisms that share similar host niches and previously known to be isolated from the same medical devices [32, 33]. Indeed, using different mouse models, active or passive immunization with the Hyr1p target, protected mice from *A*. *baumannii* infections. Interestingly, as was observed for Als3 and homologues in *S*. *aureus* [42], there are relatively low degrees of sequence identity between Hyr1 and the identified template proteins. Nonetheless, strong protective efficacy in mice was seen in cross-kingdom immunization studies of Als3p vs. *S*. *aureus* [39, 40] and with Hyr1p vs *A*. *baumannii* in this study. The observed protection among cross-kingdom antigens are likely due to highly conserved B cell epitopes given the nature of high degree of 3-D structural homology [38].

Homology modeling identified striking similarity in 3-D structures common to Hyr1p and FhaBp. Most notable among these shared structures were peptide 5 from Hyr1 of *C*. *albicans*, and its counterpart motif in the FhaBp of *A*. *baumannii*. Interestingly, our *in vivo* passive immunization studies identified antibodies targeting peptide # 5 as the most protective amongst the 8 peptide pool. These anti-Hyr1 antibodies also recognized other *A*. *baumannii* antigens including OmpA and outer membrane siderophore binding proteins. Bioinformatic analysis and computational modeling of these proteins further revealed that peptide 5 shares significant sequence identity with FhaB, OmpA and an immunoglobulin protein (Blg) that was not detected in our Western blotting analysis. Although the anti-peptide 5 antibodies reacted to FhaBp and OmpA, we do not know which of these antigens might be the targets for the protective activity seen with mice passively or actively vaccinated with Hyr1p. However, it has been previously reported that mice vaccinated with rOmpA are protected from *A*. *baumannii* bacteremia [63, 64]. The lack of detection of Blg in our Western blot studies could be attributed to conducting these analyses under conditions that are not inducing of the expression of the protein or due to technical deficiencies in isolating high molecular weight proteins (Blg is >260 kDa in mass).

Our *in vitro* studies identified two virulence mechanisms that are blocked by the anti-peptide 5 polyclonal antibody. Namely, the ability of *A*. *baumannii* to invade alveolar epithelial cells was abrogated in the presence of anti-peptide 5 antibody. In addition, this antibody appeared to have direct killing capacity of the *A*. *baumannii* study strain. Further, the bactericidal activity of the antibodies was reversed in the presence of exogenous iron, indicating that the antibody blocked iron uptake of the bacterium as part of its bactericidal mechanism. It is prudent to note that blocking of invasion cannot be attributed to the bactericidal activity of the antibody because the invasion assay is conducted over a 1 h period, while the killing assay is performed after 18–24 hours incubation.

Supporting our current findings, OmpA was reported to mediate *A*. *baumannii* invasion of epithelial cells [55]. Similarly, an OmpA deficient mutant was shown to be defective in growth under iron-limited conditions as compared to wild-type cells [58]. These results implicate OmpA in both cell adhesion and iron acquisition as virulence functions of *A*. *baumannii*. Furthermore, a TonB-dependent outer membrane siderophore that reacted to anti-peptide 5 antibodies in Western blotting assays had been previously shown to be directly involved in *A*. *baumannii* iron acquisition [59]. Finally, the hemagglutinin (FhaBp) is a known adhesion/invasin of *A*. *baumannii* [56, 57]. Although it is currently unknown if the anti-peptide 5 serum kills the bacteria by specifically binding to the OmpA, siderophore binding protein, and/or FhaBp, our data points to a putative iron starvation mechanism as potential explanation to the protective effect seen with the anti-peptide 5 antibodies *in vivo*. Studies to determine if the *in vivo* mechanism(s) of protection elicited by Hyr1p vaccination are related to these interesting *in vitro* observations are currently under investigation. Additionally, the role of OmpA, TonB-dependent outer membrane siderophore binding protein, and/or FhaBp as cross-reactive antigens to Hyr1p, and their definitive role in eliciting murine protection are currently under investigation.

Previous studies showed that *A*. *baumannii* binds to *C*. *albicans* hyphae via OmpA [35]. Our current study demonstrates that Hyr1 serves as a *C*. *albicans* receptor for OmpA through multiple lines of evidence. First, under planktonic conditions, *A*. *baumannii* was able to bind to and kill *C*. *albicans* wild-type and *hyr1/hyr1+HYR1* complemented hyphae but not *hyr1/hyr1* null mutant hyphae (Fig 3A). Second, anti-Hyr1p antibodies blocked the ability of *A*. *baumannii* to bind to *C*. *albicans* hyphae (Fig 3B). Third, *A*. *baumannii* is able to bind to and develop mixed species biofilm with *C*. *albicans* wild-type and *hyr1/hyr1+HYR1* complemented cells but not with *hyr1/hyr1* deletion mutant (Fig 3D and 3E). Finally, *C*. *albicans* wild-type or *hyr1/hyr1+HYR1* complemented strains were able to bind OmpA as detected by affinity purification assays, while the *hyr1/hyr1* mutant displayed significantly reduced OmpA binding (Fig 4). This interaction appears to be clinically significant given the recent report that *Candida* species airway colonization together with *A*. *baumannii*, during ventilator-associated pneumonia (VAP) are common among ICU patients. In fact, *Candida* species airway colonization is identified as an independent risk factor for development of *A*. *baumannii* VAP [62], as well as *P*. *aeruginosa* VAP [65].

Our findings collectively highlight the potential of using Hyr1 directed antibodies as therapeutic strategies targeting *A*. *baumannii* infections including in settings of medical devices where biofilm formation is prominent. Importantly, the antibodies lowered the concentrations of currently-used antibiotics needed to impair growth of the bacterium, including those which are deemed ineffective against XDR *A*. *baumannii* (i.e. imipenem). The antibodies were also effective in mitigating mixed species biofilms, resulting in an additive reduction in organism burden in combination with increased susceptibility to antimicrobial agents. Although these findings are yet to be confirmed in an *in vivo* model of infection, they provide strong rationale for the combined use of active or passive immunotherapy with antibiotics in treating life-threatening *A*. *baumannii* infections.

A scarcity of novel anti-*Acinetobacter* agents in the development pipeline, an escalating population of individuals at risk for *Acinetobacter* infections, and the emergence of XDR, and in some cases strains pan-resistant to all known antibiotics [66, 67], increasingly threaten global and personal health. Thus, novel immunotherapeutic approaches to reduce the incidence and severity, or enhance successful treatment of infections caused by such organisms, are highly attractive and likely to yield significant reductions in morbidity and mortality. Moreover, such approaches would be expected to decrease overall use of antibiotics, in turn reducing pressures that select for resistance. Finally, the current studies reinforce the innovative application of convergent immunity [68] to enhance the efficacy of anti-infective vaccines and immunotherapies targeting highest-priority pathogens that are increasingly resistant to conventional antimicrobial agents.

## Materials and methods

### Organism and culture conditions

The following bacterial strains were used in the study: *A*. *baumannii* HUMC1, HUMC6, and HUMC12 –all are XDR clinical strains resistant to all antibiotics except colistin; *P*. *aeruginosa* PA01 (a MDR wound isolate) [69]. Wild-type *C*. *albicans* strains SC5314 and SN250 were used in this study and have been previously described [70, 71]. The *hyr1/hyr1* mutant, and *hyr1/hyr1*+*HYR1* complemented strains were made from SN250 [71] while *als3/als3* mutant was originated from SC5314 [72]. *C*. *albicans* and *A*. *baumannii* were grown overnight in Yeast, peptone, (2%) dextrose (YPD) medium, and in Brain heart infusion broth (BHI), respectively. For hyphal development under planktonic conditions, *C*. *albicans* overnight culture was washed and inoculated at a concentration of 1x10^6^ cells/ml (determined by counting using a hemocytometer) into a pre-warmed 50:50 mixture of YPD:BHI at 37°C, for 2 h. Bacterial counts were determined using McFarland standard using optimal density at 600 nm.

### rHyr1p-N production

6x His tagged *C*. *albicans* rHyr1p-N was produced in *E*. *coli* and purified by Ni-agarose affinity column as previously described [49]. Endotoxin was removed from rHyr1p-N using ProteoSpin Endotoxin Removal kit (Norgen Bioteck Corporation, Ontario, Canada), and the endotoxin level was determined with Limulus Amebocyte Lysate endochrome (Charles River Laboratories), per manufacturer’s instruction. Using this procedure, endotoxin was reduced to <0.1 EU per dose of the vaccine.

### Planktonic co-culturing

*C*. *albicans* and *A*. *baumannii* were cultivated together (2:1 ratio) under similar growth settings for 2 h. In certain experiments, the organisms were co-cultured or grown individually, in the presence of anti-Hyr1p polyclonal antibodies (100 μg/ml). Post-incubation, the cells were either visualized by bright field microscopy, or stained with 25 μM concanavalin A (Con A)–Alexa 594 and/or 5 μM Syto 13 dyes, then imaged by Confocal Scanning Laser Microscopy (CSLM) (both dyes, Thermo Fisher Sci. Waltham, MA). Con A stains the cell wall of fungi red, and Syto 13 stains nuclei green.

### Biofilm assay

For biofilm growth, two different models were used—First, a static model: that entailed growth of organisms in 96 well microtiter plate for 24 h under non-shaking conditions, as previously described [73]. *C*. *albicans* and *A*. *baumannii* were co-cultured at 2:1 ratio (1x10^6^ cells/ml *C*. *albicans*: 5x10^5^ cells/ml *A*. *baumannii*) in the wells of the microtiter plate (100 μl final volume) for 24 h and visualized under CLSM after staining, as above. *C*. *albicans* viability in the presence and absence of *A*. *baumannii* was measured by collecting the cells from the wells, and plating dilutions on YPD plates + 5 μg/ml colistin (a concentration that kills *A*. *baumannii* HUMC1 [MIC of 2 μg/ml]). In some experiments, *C*. *albicans* were allowed to develop biofilms in the presence of *A*. *baumannii* spent medium. For this, three-day-old culture of BHI-grown *Acinetobacter* was centrifuged and filter sterilized, and concentrated by ethyl acetate followed by evaporation. The dried residue was dissolved in RPMI medium and used for *C*. *albicans* biofilm development. The extent of biofilm growth was compared to *C*. *albicans* grown in RPMI alone, and the overnight-grown biofilm activity measured by the XTT assay, by following a previously published protocol [73].

Biofilms were also developed using a simple flow biofilm model. In this system, cells adhered to silicone strips are allowed to proliferate under continuous flow of fresh medium [74]. For mixed species biofilm cultivation, a suspension of *C*. *albicans* cells (5x10^6^ cells/ml) in 50:50 YPD:BHI medium, was layered on top of the strip, and incubated for three hours at 37°C, to promote adhesion. Next, the suspension was decanted and the strip harboring adhered fungal cells were layered with a culture of *A*. *baumannii* for another two hours. One set of strips were adhered with *C*. *albicans* alone, as a comparative control. The strips were then introduced into the flow chamber, and media made to flow over the strip at the rate of 500 μl/min. After 24 h, the viability of *C*. *albicans* in the biofilm was quantified by cutting the strips into equal sizes (0.5 cm), vortexed vigorously, sonicated for 5 s at setting 3, diluted and plated on YPD containing colistin as above. The cells were also teased out, stained with 5 μM Syto 13 for 10 minutes and visualized under CLSM.

### Flow cytometry analysis

Log phase bacterial cells were incubated for 1 h with 3 or 30 μg/ml of pooled serum raised against 8 peptides of Hyr1p that is predicted to be antigenic and surface exposed. The cells were washed and counter stained with 10 μg/ml anti-rabbit IgG conjugated to Alex 488 (Thermofisher Scientific) prior to determining the binding capacity of the antibodies by using a FACSCalibur (Becton Dickinson) instrument equipped with an argon laser emitting at 488 nm. Fluorescence data were collected with logarithmic amplifiers. The population % fluorescence of 10^4^ events was calculated using the CellQuest software.

### Cell membrane preparations, Western blotting, 2-dimensional gel imaging and protein identification

*A*. *baumannii* HUMC1 membrane preparations were produced as described before [75, 76]. Briefly, the bacterium was grown overnight at 37°C with shaking in BHI. Cells were passaged in fresh medium for 3 h (log phase) at 37°C with shaking, washed, and the resultant pellet was resuspended in disintegration buffer (7.8 g/L NaH_2_PO_4_, 7.1 g/L Na_2_HPO_4_, 0.247 g/L MgSO_4_ 7.H_2_O + protease inhibitor mix (GE Healthcare) + nuclease mix (GE Healthcare) and sonicated on ice for 3x for 5 min each with the unbroken cells separated by centrifugation at 1,500 *g*. The supernatant was centrifuged for 30 min at 4°C at 4,500 rpm and was passed through a 0.45 μM filter to remove any additional cell debris. An equal volume of ice-cold 0.1 M sodium carbonate (pH 11) was added to the resulting supernatant and the mixture was stirred slowly overnight, on ice. Membrane proteins were collected by ultracentrifugation at 100,000 *g* for 45 min at 4°C, and the membranes were re-suspended in 500 ml water. Finally, the protein extract was processed with a 2-DE Cleanup Kit (Bio-Rad).

Two dimensional SDS/10%-PAGE gels of membrane preparations were used to separate proteins by size and isoelectric focusing (IEF), as described [77, 78]. For isoelectric focusing (IEF), the Bio-Rad-PROTEIN IEF system was used with 4–7 pH gradient strips (ReadyStrip IPG strips, Bio-Rad). Proteins were solubilized in 8 M urea, 2% (w/v) CHAPS, 40 mM DTT and 0.5% (v/v) corresponding rehydrated buffer (Bio-Rad). The strips were rehydrated overnight and underwent electrophoresis at 250 V for 20 min, 4000 V for 2 h, and 4,000 V for 10,000 V-h, all at room temperature. Prior to the second dimension (SDS-PAGE), the focused IPG strips were equilibrated with buffer I and II for 10 min (ReadyPrep 2-D Starter Kit, Bio-Rad). The proteins were separated on 8–16% Criterion precast Gel (Bio-Rad) and transferred to immune-Blot PVDF membranes (Bio-Rad). Membranes were treated with Western Blocking Reagent (Roche) overnight and probed with pre-immune or anti-peptide 5 serum. Membranes were washed and incubated with secondary, HRP-conjugated goat anti-rabbit IgG (Santa Cruz Biotech). After incubation with SuperSignal West Dura Extended Duration Substrate (Pierce), signals were detected using a CCD camera. The candidate band from SDS-PAGE was cut and microsequenced using MALDITOF MS/MS (UCLA Molecular Instrumentation Center) as previously described [63]. The resulting MS/MS spectra was searched against the *A*. *baumannii* strain ATCC 17978 database [63].

### Murine studies

Male BALB/c or CD-1 mice were used for all experiments. Diabetes was induced by intraperitoneal injection of 210 mg/kg streptozotocin in 0.2 ml citrate buffer 10 days prior to infection. Glycosuria and ketonuria were confirmed in all mice 7 days after streptozotocin treatment, as previously described [51, 79]. Neutropenia was induced by intraperitoneal injection of cyclophosphamide (200 mg/kg) and subcutaneous administration of cortisone acetate (250 mg/kg) on days -2 and +3, relative to infection [51]. For the hematogenously disseminated model, mice were infected intravenously with 5 x 10^7^ cells in 0.2 ml phosphate buffered saline (PBS) of log phase cells of *A*. *baumannii* HUMC1 or *P*. *aeruginosa* PA01. For the pneumonia model, mice were infected by aerosolizing bacterial cells in an inhalational chamber through a nebulizer as we previously described [51]. Briefly, mice were introduced to a Plexiglas exposure chamber (South Bay Plastics) prior to aerosolizing a 12 mL suspension of bacteria cells (1.0 x 10^11^ cells/mL) via a small-particle nebulizer (Hudson Micro Mist; Hudson RCI) driven by compressed air at 100 lb/in^2^ [80]. A standard exposure time of 1 h was used for all experiments to allow time for complete aerosolization and uniform exposure of the mice. To determine the inhaled inoculum, three mice from each experiment were sacrificed immediately after the procedure and their lungs collected and quantitatively cultured on tryptic soy agar (TSA) plates. For survival experiments, mice were followed for at least 20 days, while for tissue bacterial burden, mice were sacrificed at Day +3, relative to infection. Target tissues were harvested and bacterial burden enumerated by quantitative culturing of colony forming units (CFU).

### Active and passive immunization

Mice were vaccinated subcutaneously with 30 μg of rHyr1p-N in PBS mixed with 0.1% aluminum hydroxide (alum; Brenntag Biosector) on Day 0, boosted with a similar dose on Day +21, made diabetic on Day +25 prior to infecting them on Day +35 intravenously as described above. Control mice were vaccinated similarly with PBS alone mixed with alum. For passive immunization, diabetic or neutropenic mice were treated with a single dose of pooled anti-Hyr1p antibodies or antibodies raised against the individual peptides either 2 h prior (prophylactic) or a day after infecting the mice (therapy). In the therapy experiment, a repeat dose was administered 8 days following the infection. The doses of the antibodies are indicated in the figure legends.

### Killing assay

Bacterial cells (1 x 10^5^ cells) in Mueller Hinton II (MHII) medium were incubated in 96-well plate at 37°C for 20 h with varying concentrations of the immune serum in the presence or absence of 30-300 μM FeSO_4_. Killing activity of anti-peptide 5 serum was enumerated by CFU following sonication, and the results expressed as % killing relative to cells incubated without any anti-peptide 5 serum.

### Susceptibility of *C*. *albicans* and *A*. *baumannii* biofilms to anti-microbial agents

Susceptibility testing of anti-peptide 5 serum and/or antibiotics, or their combination, were performed in 96-well microtiter plates. *A*. *baumannii* (1x10^5^ cells/100 μl) were treated with serum alone (12.5%/well), colistin alone (concentrations ranging from 2–0.125 μg/ml) or a combination of serum and different concentrations of colistin. Some wells that contained only bacterial cells, free of any treatment were included as controls. The plates were incubated at 37°C for 16 h, and turbidity in each well measured spectrophotometrically at OD 600. The same protocol was utilized for testing another drug, imipenem (concentrations ranging from 32–2 μg/ml). For a set of experiments, serum, antibiotics and their combination, were used against mixed species biofilms, and their impact was measured at OD 600.

To evaluate the potential enhanced activity of colistin in combination with anti-peptide 5 serum against *A*. *baumannii in vitro* (time kill curves), 1x10^5^ bacterial cells [1×10^6^ colony-forming units (CFU)/ml] were transferred into wells of a 96-well plate containing MH medium with 0.5X, 0.25X and 0.125X MIC of colistin (1 μg/ml, 0.5 μg/ml, 0.25 μg/ml) or a combination of the individual concentrations of colistin with 12.5% serum and incubated at 37°C. Inoculated MH medium without drug/serum served as controls. Aliquots were obtained at 0, 2, 4, 8, 12 and 24 h for quantification and data presented as CFU versus time.

### Biotin labeling of bacterial membrane proteins, affinity purification and protein identification

*A*. *baumannii* membranes were prepared as above. The cell membrane protein extracts were biotin labeled by incubation with Ez-Link Sulfo-NHS-LS Biotin (0.5 mg/ml; Pierce) for 15 minutes at 37°C. Pre-germinated short hyphae (1 x 10^6^) of *C*. *albicans* SC5314, *C*. *albicans hyr1/hyr1*, *C*. *albicans hyr1/hyr1*+*HYR1* complemented, or *C*. *albicans als3/als3* were incubated for 1 h on ice with 250 μg biotin-labeled *A*. *baumannii* cell surface proteins in PBS plus 1.5% *n*-octyl-β-d-glucopyranoside and protease inhibitors. Unbound proteins were washed away by three rinses with the same buffer. The *Acinetobacter* cell proteins that remained bound to the fungal cells were eluted twice with 6M urea (Fluka), and the proteins separated on 8–16% SDS-PAGE and transferred to PVDF-plus membranes (GE Water & Process Technologies). The membrane was treated with Western Blocking Reagent (Roche) and probed with a 1:1000 dilution of anti-biotin antibody (Abcam, Cambridge, MA), followed by incubation with SuperSignal West Dura Extended Duration Substrate (Pierce). Some blots were also probed with 1:1000 diluted anti-*E*. *coli* OmpA antibodies (Antibody research Co., St. Peters, MO) and signals detected using a CCD camera. Protein bands of interest were excised and identified by MALDI-TOF—MS/MS as above.

### Computational modeling of structural homology

Complimentary homology and energy-based modeling algorithms were conducted to characterize and compare the overall physicochemical and structural features of *C*. *albicans* Hyr1p. Further, these two protocols were used to prioritize potential structural domains that may serve as epitopes for cross reactivity of anti-Hyr1 antibody. While both protocols involve the use of homology-based threading algorithms, initial studies made use of the Phyre2 modeling software package [81] that prioritizes remote template detection, alignment, 3-D modeling and ab initio protocols. Model refinement was carried out using the iTasser server [82, 83] which utilizes a meta-threading approach to identify PDB templates which are then assembled into continuous domains using replica-exchange Monte Carlo simulations and ab initio modeling. Notably, the iTasser server has consistently ranked as a top homology modeling application and was ranked as the top free modeling protocol in a recent independent modeling study [84]. As a confirmatory measure, additional stochastic modeling was carried out using the Quark server [83]. Select regions of resulting comparative homologues were then subjected to 3-D alignment to identify areas of greatest homology using the Smith-Waterman [85] algorithm as implemented within Chimera [86]. Sequence alignments to identify putative shared epitopes between Hyr1 and other proteins were carried out using CLUSTALW [87].

### Ethics statement

All procedures involving mice were approved by the IACUC of the Los Angeles Biomedical Research Institute at Harbor-UCLA Medical Center (Protocol number 20295), according to the NIH guidelines for animal housing and care. Moribund mice according to detailed and well-characterized criteria were euthanized by pentobarbital overdose, followed by cervical dislocation.

### Statistical analysis

The nonparametric log-rank test was used to determine differences in survival times. The nonparametric Wilcoxon rank sum test was used to analyze differences in tissue bacterial burden, the ability of anti-peptide 5 to affect invasion of host cells, to kill *A*. *baumannii*, or to assess the effect of combination treatment of the anti-peptide 5 sera with antibiotics on bacterial survival and biofilm formation. For all comparisons, a *P* value < 0.05 was considered significant.

## Supporting information

S1 Fig Binding Assay of purified IgG (100 μg/ml) raised against Hyr1p 8 peptides to XDR *A*. *baumannii* clinical strains with known clonal variability. (TIF)

S2 Fig Cell free extract of *A*. *baumannii* prevents *C*. *albicans* (CA) filamentation and biofilm formation (A). *C*. *albicans* cells have higher viability in a mixed flow biofilm model versus the mixed static biofilm model (B).
(TIF)

S3 Fig Time kill curves of *A*. *baumannii* by sub-inhibitory concentrations of colistin, anti-peptide 5 serum or a combination of both. Colistin was used at 1, 0.5, or 0. 25 μg/ml representing (0.5, 0.25, or 0.125 MIC, while anti-peptide 5 serum was used at 12.5% representing 0.5 MIC.
(TIFF)
