# Supplementary figures and images for: The Hyr1 protein from the fungus Candida albicans is a cross kingdom immunotherapeutic target for Acinetobacter bacterial infection
Source: PLoS Pathog. 2018 May 10;14(5):e1007056. doi: 10.1371/journal.ppat.1007056 (PMC5963808; doi:10.1371/journal.ppat.1007056)

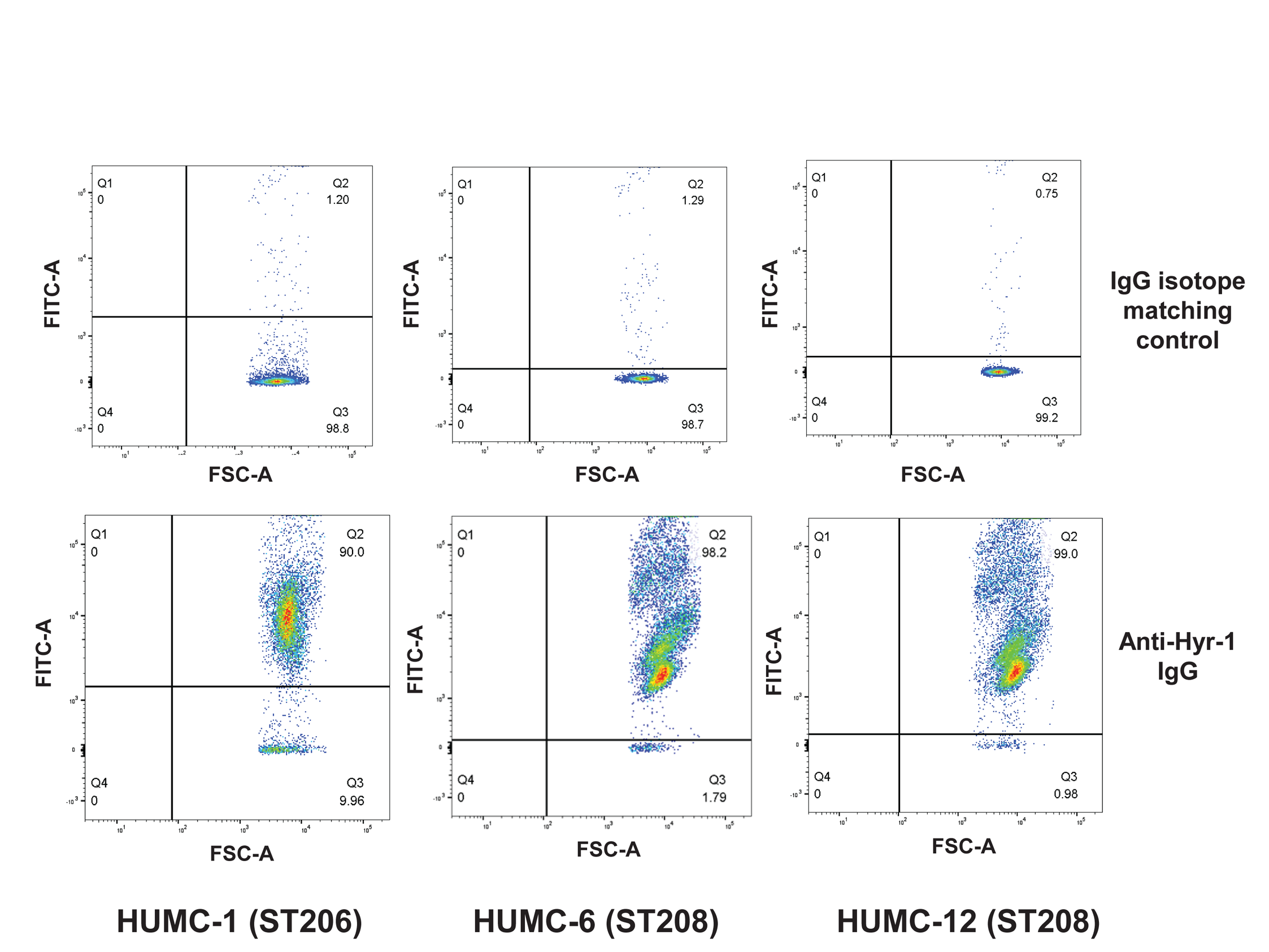

Supplement: S1 Fig — (TIF) [file ppat.1007056.s001.tif]

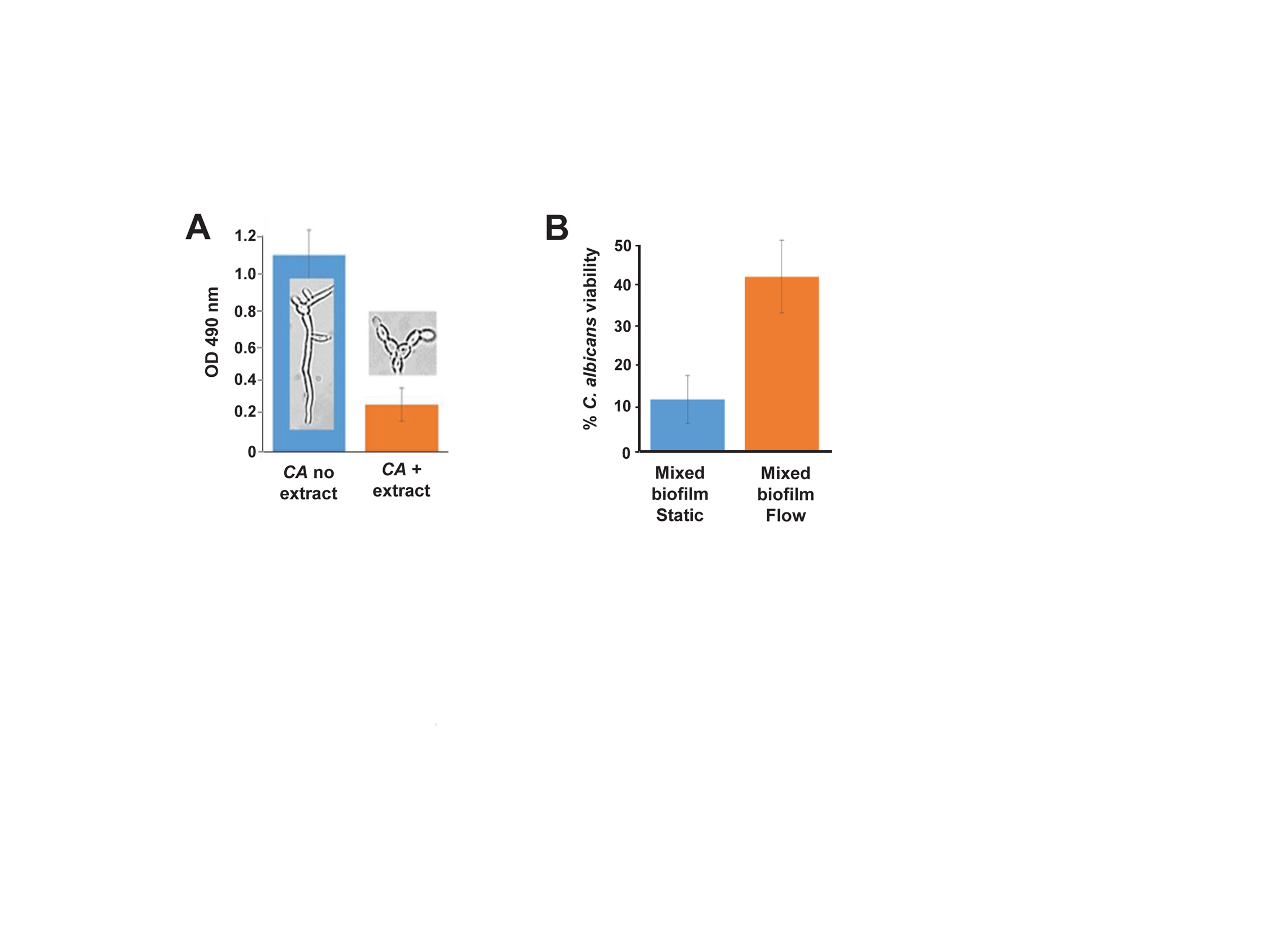

Supplement: S2 Fig — Cell free extract of A. baumannii prevents C. albicans (CA) filamentation and biofilm formation (A). C. albicans cells have higher viability in a mixed flow biofilm model versus the mixed static biofilm model (B). (TIF) [file ppat.1007056.s002.tif]

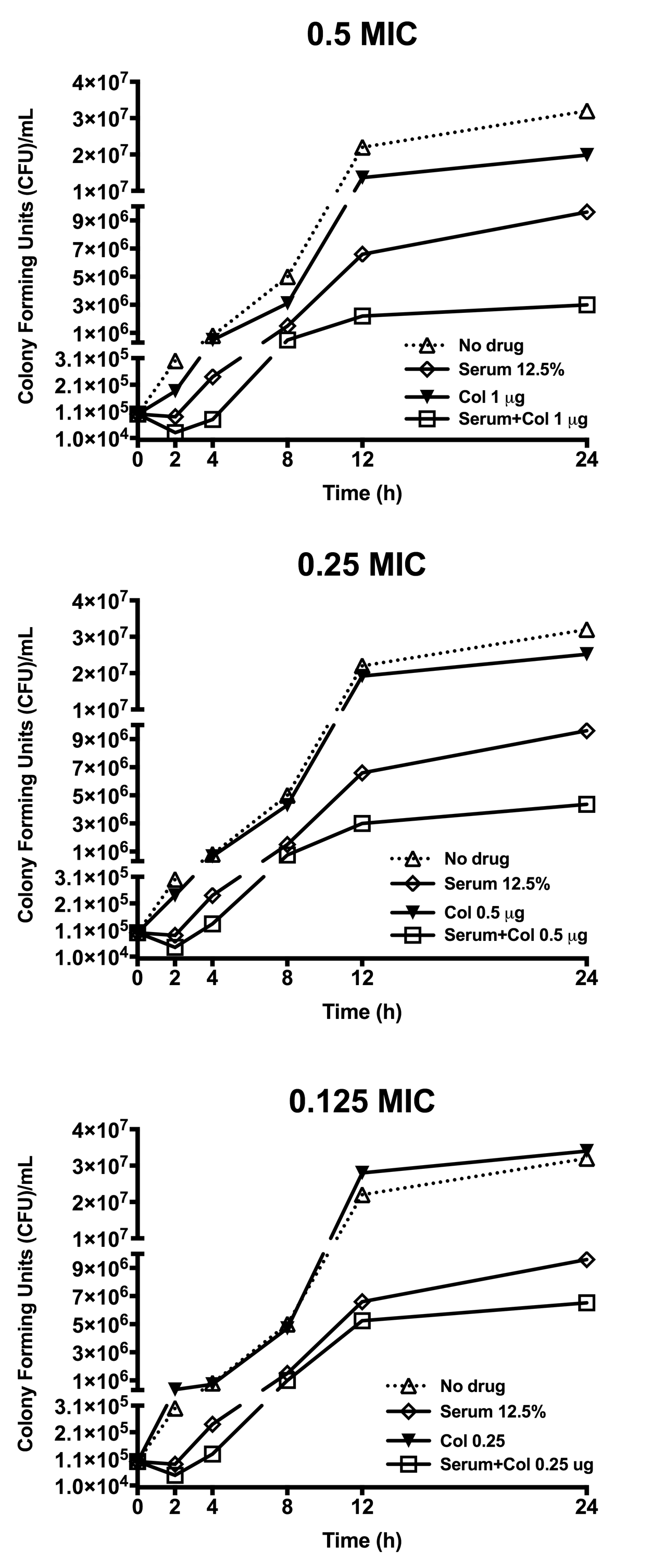

Supplement: S3 Fig — Colistin was used at 1, 0.5, or 0. 25 μg/ml representing (0.5, 0.25, or 0.125 MIC, while anti-peptide 5 serum was used at 12.5% representing 0.5 MIC. (TIFF) [file ppat.1007056.s003.tiff]
